# Acute Hippocampal Damage as a Prognostic Biomarker for Cognitive Decline but Not for Epileptogenesis after Experimental Traumatic Brain Injury

**DOI:** 10.3390/biomedicines10112721

**Published:** 2022-10-27

**Authors:** Eppu Manninen, Karthik Chary, Riccardo De Feo, Elina Hämäläinen, Pedro Andrade, Tomi Paananen, Alejandra Sierra, Jussi Tohka, Olli Gröhn, Asla Pitkänen

**Affiliations:** A. I. Virtanen Institute for Molecular Sciences, University of Eastern Finland, P.O. Box 1627, FI-70211 Kuopio, Finland

**Keywords:** epilepsy, traumatic brain injury, magnetic resonance imaging, cognitive dysfunction, hippocampus

## Abstract

It is necessary to develop reliable biomarkers for epileptogenesis and cognitive impairment after traumatic brain injury when searching for novel antiepileptogenic and cognition-enhancing treatments. We hypothesized that a multiparametric magnetic resonance imaging (MRI) analysis along the septotemporal hippocampal axis could predict the development of post-traumatic epilepsy and cognitive impairment. We performed quantitative T_2_ and T_2_* MRIs at 2, 7 and 21 days, and diffusion tensor imaging at 7 and 21 days after lateral fluid-percussion injury in male rats. Morris water maze tests conducted between 35–39 days post-injury were used to diagnose cognitive impairment. One-month-long continuous video-electroencephalography monitoring during the 6th post-injury month was used to diagnose epilepsy. Single-parameter and regularized multiple linear regression models were able to differentiate between sham-operated and brain-injured rats. In the ipsilateral hippocampus, differentiation between the groups was achieved at most septotemporal locations (cross-validated area under the receiver operating characteristic curve (AUC) 1.0, 95% confidence interval 1.0–1.0). In the contralateral hippocampus, the highest differentiation was evident in the septal pole (AUC 0.92, 95% confidence interval 0.82–0.97). Logistic regression analysis of parameters imaged at 3.4 mm from the contralateral hippocampus’s temporal end differentiated between the cognitively impaired rats and normal rats (AUC 0.72, 95% confidence interval 0.55–0.84). Neither single nor multiparametric approaches could identify the rats that would develop post-traumatic epilepsy. Multiparametric MRI analysis of the hippocampus can be used to identify cognitive impairment after an experimental traumatic brain injury. This information can be used to select subjects for preclinical trials of cognition-improving interventions.

## 1. Introduction

Annually, 3.5 million people in the USA, 2.5 million people in Europe, and 69 million people globally suffer a traumatic brain injury (TBI) [1,2]. A TBI leads to post-traumatic epilepsy (PTE) in 1.3% to 53% of patients [3]. It also evokes a range of cognitive impairments (CI), including deficiencies in learning, as well as deficits in both short- and long-term memory [4]. A large number of proof-of-concept trials have demonstrated that it is possible to achieve treatment effects with antiepileptogenic, neuroprotective as well as behavior or cognition-improving compounds in animal models of TBI; nonetheless, none of these antiepileptogenic or recovery-enhancing treatments have successfully passed through clinical trials [5,6]. One major reason for this dismal situation is the lack of epilepsy biomarkers that would allow efficient stratification of patients, thus decreasing the cost of clinical trials [7].

Magnetic resonance imaging (MRI) is a safe imaging method [8], enabling repeated in vivo investigations of the evolving pathologies evoked by a TBI [9]. For this reason, diagnostic and prognostic MRI biomarkers of epileptogenesis are being actively investigated in preclinical and clinical settings [10,11].

A lateral fluid-percussion injury (FPI) in rodents is a model of TBI and PTE [12,13]. It evokes several cellular changes in the hippocampus proper and the dentate gyrus, including a loss of principal cells and interneurons, mossy fiber sprouting, vascular abnormalities, and gliosis. These changes have been proposed to underlie the evolution of hyperexcitability and the subsequent cognitive decline [14,15,16]. Typically, abnormalities have been detected as being located ipsilateral to the injury, but some investigators have also identified a milder injury in the contralateral hippocampus [15,17]. Importantly, in different animal models of epilepsy, some hippocampal pathologies have shown promise as biomarkers of epileptogenesis [18,19,20,21]. However, the diagnostic accuracy of most of these findings remain to be explored in more detail.

In an effort to expand on the previous observations in a larger animal cohort, we investigated the hypothesis that early quantitative T_2_, T_2_*, and diffusion tensor imaging (DTI) MRI measures in different parts of the septotemporal hippocampal axis would be able to predict the development of CI or PTE after a lateral FPI. The different quantitative MRI measures that were used offer complementary information on the evolving tissue pathology and were also used in regularized multivariable logistic regression analyses. The multivariable approach may help to elicit diagnostic information from the complex ongoing pathophysiological changes that occur in the hippocampus after the TBI that would be inaccessible to single-variable approaches. The analysis was performed at different locations along the septotemporal axis of the hippocampus, and thus accounted for the possible spatial dependence of hippocampal function or pathology along this axis. As a severe lateral FPI causes an expansion of the lateral ventricles and evokes hippocampal atrophy, it decreases the accuracy of standard image registration procedures. Thus, in order to conduct an accurate between-subject comparison of quantitative MRI parameters within the hippocampus, we utilized convolutional neural network-based segmentations of the hippocampi to improve the accuracy of the image registrations.

## 2. Materials and Methods

### 2.1. Study Design

**Figure 1:**
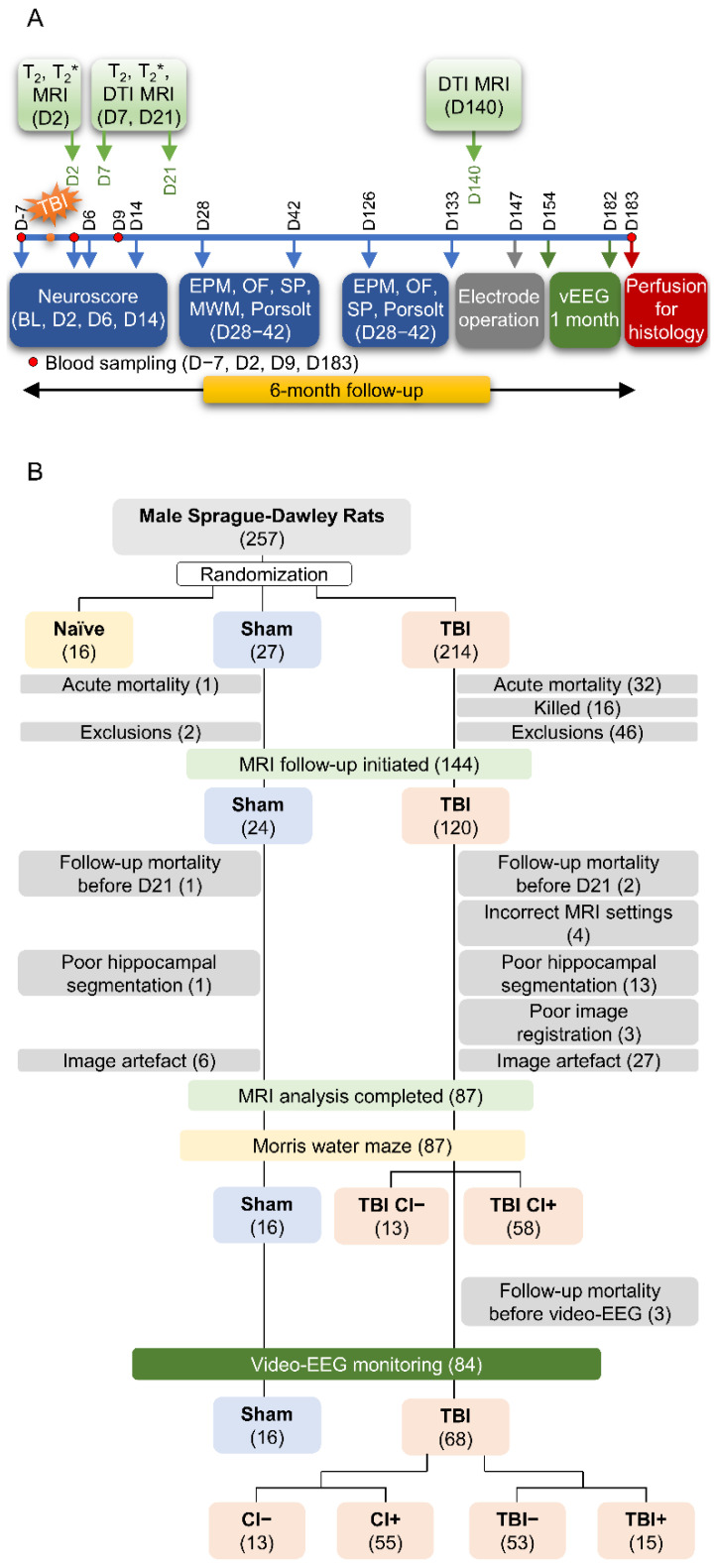
Study flow, number of animals, and exclusions. (**A**) Study design for a 6-month follow-up after experimental traumatic brain injury (TBI). (**B**) Exclusions. A total of 257 male rats were included in the study. The total was divided into 8 subsequent subcohorts over a period of three years as the capacity of the video-EEG monitoring unit was limited to 30 animals. After acute and follow-up mortality and exclusions, 24 sham-operated rats and 120 rats with lateral fluid-percussion-induced TBI were included in the MRI follow-up. The severity of cognitive impairment was assessed using the Morris water maze test (MWM) on day (D) 35-D39. The development of epilepsy was determined by undertaking continuous 30-d video-EEG monitoring on the 6th post-injury month [22]. The reasons for MRI-related exclusions included missing data (follow-up mortality), incorrect MRI settings, poor hippocampal segmentation, poor image registration, and presence of image artefacts. Thus, data from 16 sham and 68 TBI animals were included in the final analysis. Of the TBI animals, 55 displayed a cognitive impairment and 15 had post-traumatic epilepsy. Abbreviations: CI−, rats without cognitive impairment; CI+, rats with cognitive impairment; T_2_, T_2_ relaxation time; T_2_*, T_2_* relaxation time; DTI, diffusion tensor imaging; EPM, elevated plus-maze; MRI, magnetic resonance imaging; OF, open-field; SP, sucrose preference; TBI−, rats without epilepsy; TBI+, rats with epilepsy.

Study design, animal numbers, and exclusions related to the present analysis are summarized in Figure 1. The data derives from the large EPITARGET rat cohort (*n* = 257, male) that has been described in detail before [22,23]. Due to imaging or image-analysis-related challenges, only 87 of the 144 (60%) MRI-imaged rats were included in the analysis of the hippocampus (16 sham, 71 TBI).

#### 2.1.1. Diagnosis of Post-Traumatic Epilepsy

At five months post-TBI, on day (D) 147, the rats were anesthetized and implanted with three skull electrodes. Starting on D154 (one week after electrode implantation), rats underwent a continuous (24/7) video-EEG monitoring for 4 weeks to diagnose the presence of post-traumatic epilepsy (for details, see [22]). Rats were defined as having epilepsy if at least one unprovoked electrographic seizure was detected. A seizure was defined as a high-amplitude rhythmic discharge, representing an atypical EEG pattern (repetitive spikes, spike-and-wave discharges, poly-spike-and-wave, or slow-waves, frequency and amplitude modulation), that lasted longer than 10 s [13]. The prevalence of epilepsy in the entire EPITARGET cohort was 27% (31/115) and in the subcohort of the 68 TBI animals included in the present analysis 22% (15/68).

#### 2.1.2. Cognitive Impairment

The Morris water maze test was performed on D35–39 after the injury to assess spatial learning and memory (for details, see [22]). Briefly, the test started with a habituation day (D0), during which the rat was allowed to habituate to the maze during a 60 s swimming trial (no platform present). During the following 3 d (D1–3 of acquisition learning), the rat was placed into the pool at 1 of the 4 designated starting points (South, West, North, East), facing the wall. Each rat underwent 5 consecutive trials per day (60 s/trial) for 3 d. If the rat did not find the platform within 60 s, it was gently guided to the platform by the experimenter. At the end of testing on D3, a probe trial was performed without the platform present. The probe trial was repeated on D5. As outcome measures, we recorded (1) path length (swimming distance) for each trial (cm), (2) swimming speed (velocity, cm/s), (3) time to reach the platform (latency, s), (4) latency to the platform zone in probe trials on D3 and D5, (4) learning speed on D1-D3 (the difference between the escape latency in trials 1 and 5, normalized to the escape latency in trial 1 on each testing day) [21], and (5) speed of forgetting (the escape latency measured during the last training session of each day vs. the escape latency measured during the first session of the subsequent day) [21]. The outcome parameters were obtained using EthoVision^®^ XT (v. 7.1, Noldus Information Technology, Wageningen, The Netherlands).

The cut-point analysis of Morris water maze data of the entire EPITARGET animal cohort, including 23 sham-operated and 118 rats with TBI, was performed to identify the best parameter that could be used to differentiate cognitively impaired (CI+) from non-impaired (CI−) animals [22]. The analysis revealed that the latency cut-off value of 19.2 sec to reach the platform on the third testing day (D37 post-TBI) distinguished TBI animals from sham-operated experimental controls with an AUC value of 0.94 (84% sensitivity, 100% specificity, *p* < 0.001). In the entire EPITARGET cohort, 70% (98/141) of the rats with TBI were categorized into the CI+ and 30% (20/141) into the CI− group. In the present subcohort, 81% (55/68) of the TBI animals belonged to CI+ and 19% (13/68) to CI− groups.

All experiments were approved by the Animal Ethics Committee of the Provincial Government of Southern Finland and performed in accordance with the guidelines of the European Community Council Directives 2010/63/EU.

### 2.2. MRI Acquisition

#### 2.2.1. Equipment

The animals were anesthetized with isoflurane (1.5–2.5%; carrier gas comprising 70% N_2_, 30% O_2_) during MRI, ensuring that the breathing rate was maintained at 50–70 breaths/min. Their body temperature was monitored using a rectal probe and kept at 36–37 °C. Imaging was done using a 7-tesla Bruker PharmaScan MRI scanner (Bruker BioSpin MRI GmbH, Ettlingen, Germany) with a volume transmitter coil and a quadrature surface receiver coil designed for the rat head.

#### 2.2.2. T_2_ MRI

Twenty-four axial slices (thickness 0.50 mm) with a resolution of 0.20 × 0.20 mm^2^ were imaged using a multi-slice-multi-spin-echo pulse sequence with six echoes (echo times 14.6–87.6 ms) and repetition time 3.0 s. Two signal averages were acquired. The partial Fourier-accelerated encoding matrix size was 212 × 160, and the reconstructed image matrix size was 212 × 212. The imaging time for the sequence was 16 min 5 s. The T_2_ relaxation time was estimated in each imaging voxel using non-linear least squares.

#### 2.2.3. T_2_* MRI

The data for T_2_* relaxation time estimation was acquired using a multi-slice-multi-gradient-echo sequence. Twenty-four 0.50 mm-thick axial slices were acquired with a resolution of 0.15 × 0.15 mm^2^. Twelve equally spaced gradient echoes were acquired (echo times 4–59 ms) with a repetition time of 1.6 s and four signal averages were collected. The partial Fourier-accelerated encoding matrix size was 96 × 106, and the reconstructed image matrix size was 96 × 142. The imaging time for the sequence was 11 min 37 s.

Consecutive gradient echo images were registered to the first echo image with a rigid transformation using Advanced Normalization Tools (ANTs version 2.3.5, http://stnava.github.io/ANTs/, accessed on 6 December 2021) [24]. Then, the non-linear least squares estimation was used to estimate the T_2_* relaxation time in each imaging voxel by fitting a two-parameter monoexponential signal decay model (relaxation time T_2_*, signal intensity at zero echo time).

#### 2.2.4. Diffusion Tensor Imaging (DTI)

Diffusion-weighted images were acquired with a three-dimensional diffusion-weighted segmented spin echo echo-planar imaging sequence. Sixty diffusion-weighted (b-value 2000 s/mm^2^) and four non-diffusion-weighted images were acquired at a physical resolution of 0.15 × 0.15 × 0.50 mm^3^. Then, after eddy current and motion correction as well as diffusion volume outlier removals, the following parameters were estimated: diffusion tensor eigenvalues (λ_1_, λ_2_, λ_3_), fractional anisotropy (FA), mean diffusivity (MD), radial diffusivity (RD), and Westin parameters (linear (c_l_), planar (c_p_), and spherical (c_s_) component of the diffusion tensor).

### 2.3. MRI Analysis

#### 2.3.1. Summary

**Figure 2:**
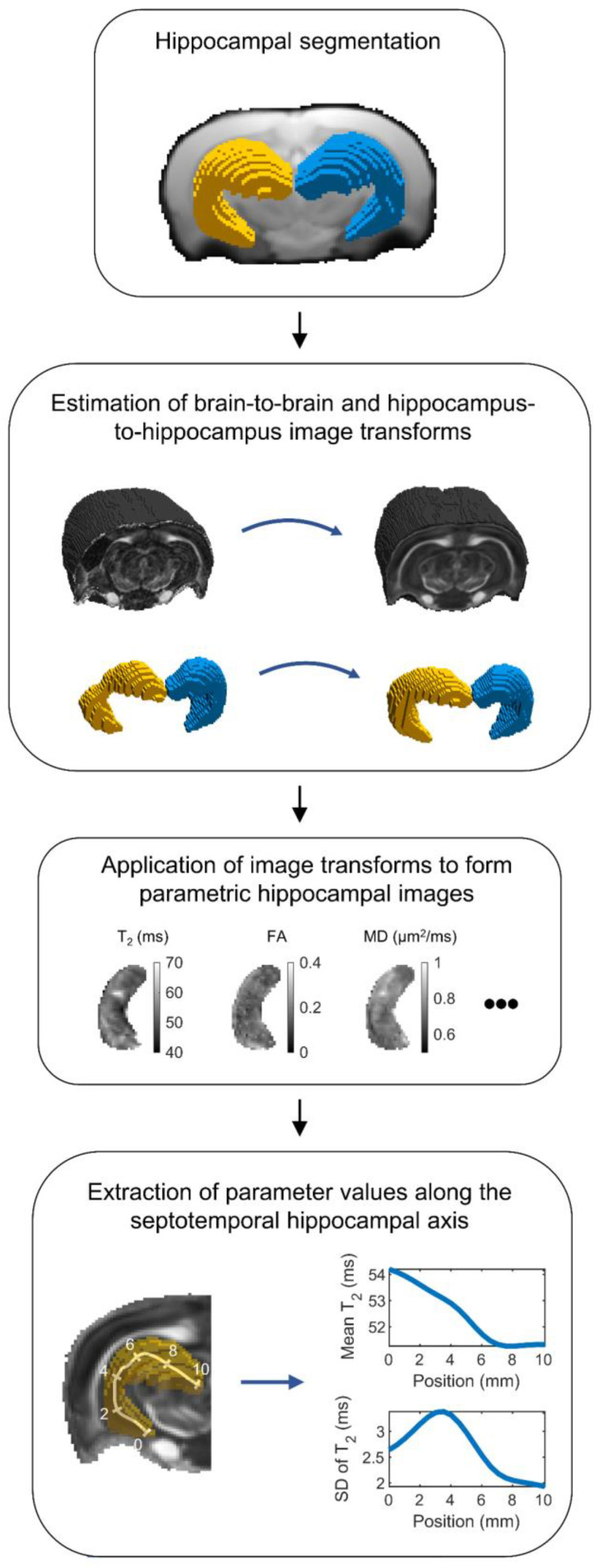
Flowchart of the MRI analysis. First, the ipsilateral and contralateral hippocampi were segmented for each animal using an automated machine learning-based segmentation method. Then, the segmented hippocampal masks were used to estimate image transformations from the hippocampus of each animal to the reference hippocampus. The estimated image transformations were then applied to MRI parameter maps to form parametric hippocampal images in the same reference space. Finally, the hippocampus was skeletonized along its septotemporal axis, and the weighted mean and standard deviation (SD) of each quantitative MRI parameter was calculated along the axis. Abbreviations: FA, fractional anisotropy; MD, mean diffusivity.

The MRI analysis is summarized in Figure 2. Briefly, convolutional neural network-based hippocampal segmentation was used to improve image registration accuracy within the hippocampi of the individual animals. Then, quantitative MRI parameter maps of each animal were transformed to the same reference space. In this reference space, each voxel within the hippocampus was projected to a location on a skeleton defining the septotemporal axis of the hippocampus. Finally, the weighted mean and standard deviation (SD) were calculated for each MRI parameter along the hippocampus.

#### 2.3.2. Hippocampal Segmentation

Due to the presence of notable atrophy and disorientation of the hippocampi of rats with TBI, the use of conventional co-registration tools would have resulted in inaccurate registrations between the hippocampi. Therefore, a convolutional neural network, termed MU-Net-R, was trained to segment the ipsilateral (left) and contralateral (right) hippocampi of each animal at all three imaging time points (D2, D7, D21 after TBI or sham-operation). MU-Net-R, as well as its training and validation, have been described earlier [23]. MU-Net-R has been shown to produce accurate hippocampal segmentations, which are not biased by the presence of a brain injury [23]. Briefly, the individual echo images of the T_2_* imaging sequence were averaged to obtain anatomical images with high T_2_*-weighted contrast and a high signal-to-noise ratio. Then, a trained investigator (E.H) manually segmented the left (ipsilateral to the lesion) and right hippocampus from MRIs of 4 TBI and 2 sham-operated rats at each time point, generating a training set of images that was used to train MU-Net-R. MU-Net-R exhibits an encoder/decoder structure and optimizes the generalized Dice loss [25] using the RAdam optimizer [26] for training. After training, MU-Net-R segmented the hippocampi in the multi-gradient-echo images of each rat at the 3 time points.

Instead of training separate neural networks for the different imaging sequences, we used image registration to transfer the hippocampal segmentation (mask) from the T_2_* sequence to the T_2_ and DTI sequences. The three sequences were matched for slice thicknesses and locations and were imaged during the same session, allowing us to perform slice-by-slice 2D image registration. Slice-by-slice registration was chosen because it outperformed 3D registration. The slices were registered with an affine and a symmetric image normalization (SyN) registration using ANTs. Then, the segmented hippocampal masks were transformed from the T_2_* images to the T_2_ and DTI images.

Hippocampal segmentations were visually verified. Animals with clear mis-segmentations were excluded from the analysis (sham: 1/23, 4.3%; TBI: 13/114, 11%). Examples of mis-segmentations are shown in Figure S1.

#### 2.3.3. Registration of Hippocampal Images

**Figure 3:**
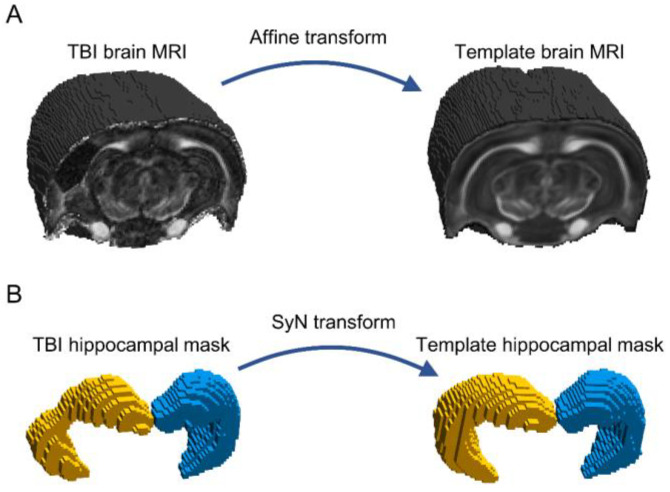
Image registration. (**A**) Each brain was registered into the whole brain sham template, using an affine registration. (**B**) Then, the segmented ipsilateral (left, yellow) and contralateral (right, blue) hippocampal masks were registered into the hippocampal template masks, using symmetric image normalization registration (SyN). The parametric MRI maps were subsequently transformed to the template space, and the contributions of non-hippocampal voxels were suppressed to create the hippocampal images.

The hippocampal segmentations were used to create hippocampal images (i.e., images from which the signals originating from outside the hippocampus were removed) registered to the same reference space. First, a template for the sham brain was formed by registering the b0 (non-diffusion-weighted) images of the DTI scans to one of the sham animals, and subsequently, the transformed images were averaged. Then, the brain image of each animal was transformed to the template brain using an affine transformation (Figure 3A). The affine transformation was applied separately to the ipsilateral and contralateral hippocampal masks, which were registered to the corresponding hippocampal masks of the template using SyN (Figure 3B). Finally, the computed affine (brain-to-brain) and SyN (hippocampus-to-hippocampus) transformations were applied to each parametric MRI map after the removal of the contribution of non-hippocampal voxels.

Image registration accuracy was inspected visually. Animals with clear mis-registrations were excluded from the analysis (sham: 0/22, 0.0%; TBI: 3/101, 3.0%). Examples of mis-registrations are shown in Figure S2.

#### 2.3.4. Parameter Extraction

**Figure 4:**
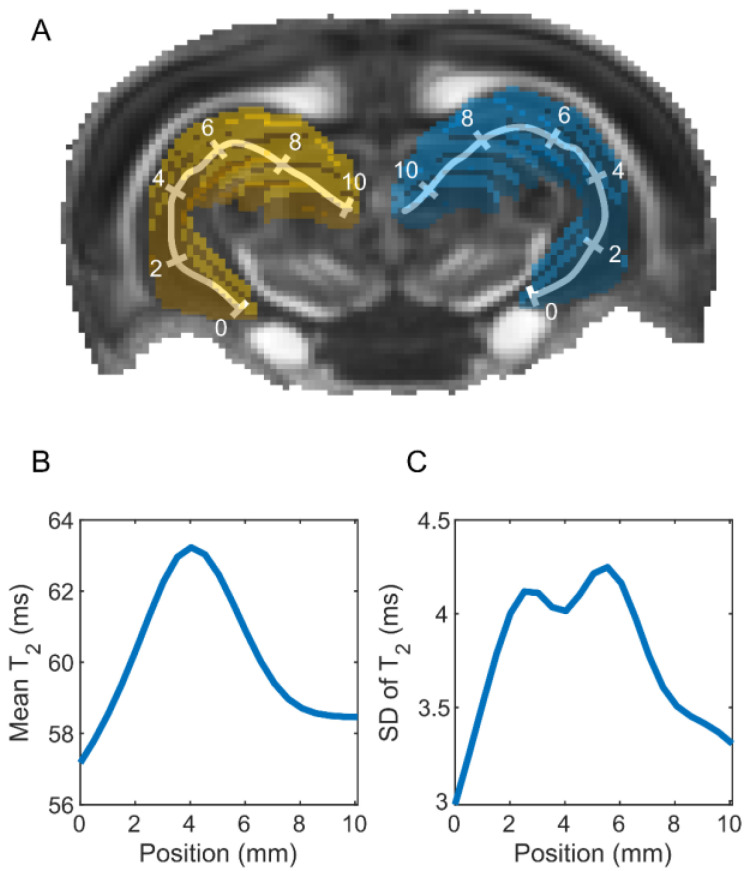
Computation of MRI metrics along the septotemporal axis of the rat hippocampus. (**A**) Ipsilateral (left, yellow) and contralateral (right, blue) hippocampus in the template brain of a sham-operated control. The left and right hippocampal skeletons are shown as polylines with 2-mm segments, in which the temporal end of the hippocampus is labeled as 0. The value of each imaging voxel within the hippocampus was projected onto the skeleton after applying a Gaussian spatial filter. (**B**) Weighted mean and (**C**) standard deviation (SD) of the T_2_ relaxation time along the hippocampal axis on day 2 after TBI for one animal. The measures were computed for each animal and MRI parameter map.

The hippocampus was skeletonized to assess hippocampal abnormalities along its septotemporal axis [27] (Figure 4A). A pixelized midline skeleton was first formed using the MATLAB (MATLAB Release 2018b, The MathWorks, Inc., Natick, MA, USA) function “bwskel”. Equidistantly positioned pixels (spacing 1 mm) on the skeleton were selected to create a continuous, parameterized skeleton using a piecewise cubic interpolating polynomial (MATLAB function “pchip”). Each voxel within the hippocampus was projected to its nearest position on the skeleton and then spatially smoothed (Gaussian filter, full width at half maximum 1.5 mm). Using the spatially smoothed voxel weights, the weighted mean (Figure 4B) and SD (Figure 4C) along the hippocampal length were computed for each animal and MRI parameter map. Voxels near the hippocampal surface were excluded to avoid the partial volume effect. In addition, voxels that exhibited image artifacts and the most caudal imaging slices with lower signal-to-noise ratios were excluded from the analyses (for details, see Supplementary Materials Figures S3 and S4).

### 2.4. Statistics

#### 2.4.1. Single-Variable Predictors

The Mann–Whitney U test (MATLAB function “ranksum”) was used to analyze whether the weighted mean or SD calculated for each MRI parameter map along the septotemporal axis of the hippocampus differed between the following groups: (a) sham and TBI, (b) TBI− (rats without epilepsy) and TBI+ (rats with epilepsy), and (c) CI− (rats without a cognitive impairment) and CI+ (rats with a cognitive impairment). The Benjamini–Hochberg procedure [28] was used to control for multiple comparisons, using an average false discovery rate of 0.05.

#### 2.4.2. Multi-Variable Regularized Logistic Regression Analysis

We performed a multi-predictor quantitative MRI analysis along the septotemporal axis of the hippocampus after experimental TBI, which was made possible by the improved registration accuracy enabled by convolutional neural network-based hippocampal segmentations. We fitted elastic net-based regularized logistic regression models to distinguish (a) sham and TBI animals, (b) TBI− (rats without epilepsy) and TBI+ (rats with epilepsy) animals, and (c) CI− (rats with no cognitive impairment) and CI+ (rats with cognitive impairment) animals. Elastic net combines least absolute shrinkage selector operator (LASSO) and ridge regularization to reduce model overfitting by forcing the coefficients of unnecessary predictor variables towards zero. We used the MATLAB implementation of Glmnet software (version 11 March 2015) for fitting the logistic regression models (https://hastie.su.domains/glmnet_matlab/, accessed on 31 October 2019) [29,30].

At each location of the hippocampus, we included all the MRI variables (mean and SD of each MRI parameter map at each time point) as predictor variables. We chose equal weighting for LASSO and ridge regularization [31]. The observations were weighted to adjust for the class imbalance, such as the difference between the number of sham and TBI animals. Model fitting was performed by minimizing the binomial deviance within a nested (externally validated) leave-one-out cross-validation framework [32]. The value of the regularization parameter was set based on the inner cross-validation loop and the evaluation was performed in the outer cross-validation loop. We computed the cross-validated area under the receiver operating characteristic curve (AUC) as a measure of goodness-of-fit using the pooling method [33]. A 95% confidence interval for the AUC was estimated using a bias-corrected and accelerated bootstrap method with 10,000 samples.

#### 2.4.3. Analysis of the Hippocampal Volume

For each animal and time point, the volume of the ipsilateral and the contralateral hippocampus was computed from the segmentation. The volumes of both the ipsilateral and the contralateral hippocampus at each time point were used as predictor variables in elastic net-regularized logistic regression models. The models attempted to distinguish (a) sham and TBI animals, (b) TBI− (rats without epilepsy) and TBI+ (rats with epilepsy) animals, and (c) CI− (rats with no cognitive impairment) and CI+ (rats with cognitive impairment) animals.

## 3. Results

### 3.1. Study Flow, Number of Animals, and Exclusions

Animals with missing data or poor segmentation, registration, or image quality were excluded from the analysis (sham: 8/24, 33%; TBI: 52/120, 43%) (Figure 1B). After the exclusions, 16 sham and 68 TBI animals were included in the analyses. Of the 68 TBI animals, 15 (22%) had post-traumatic epilepsy (TBI+), and 55 (81%) had cognitive impairment (CI+). Of the 15 rats with epilepsy, 12 (80%) belonged to the CI+ group. Thus, 58 of the 68 TBI animals had either epilepsy, a cognitive impairment, or both.

### 3.2. MRI Analysis

#### 3.2.1. Single-Parameter Differences between the Groups

##### Sham vs. TBI

**Figure 5:**
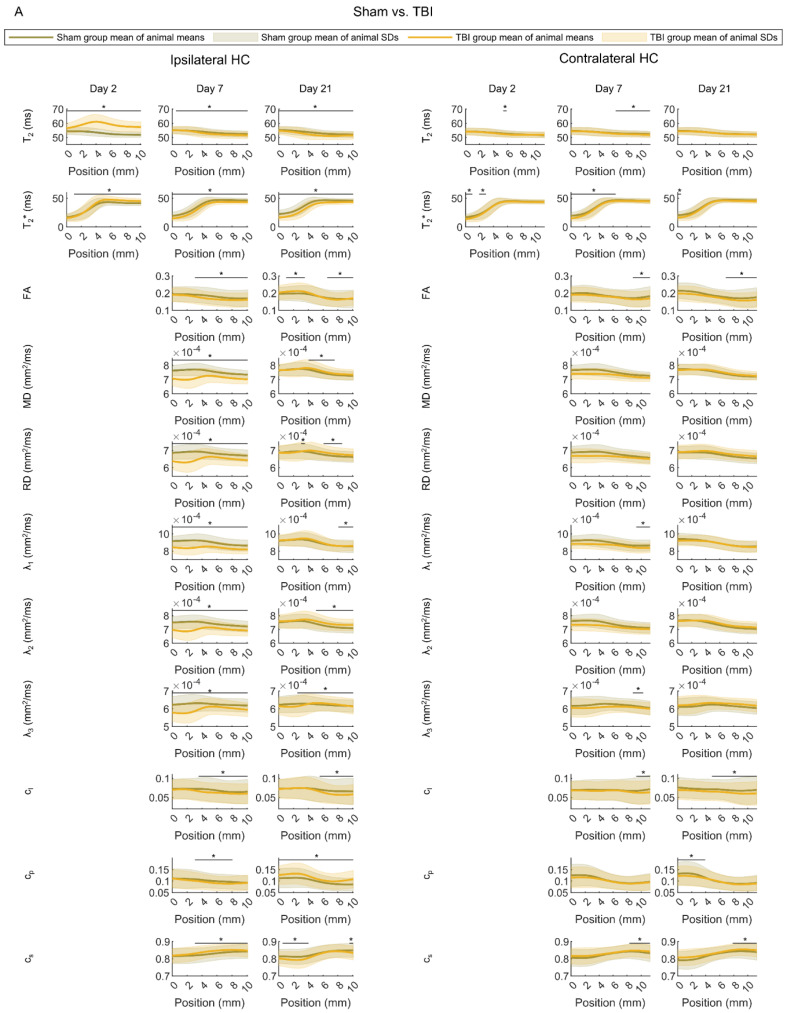

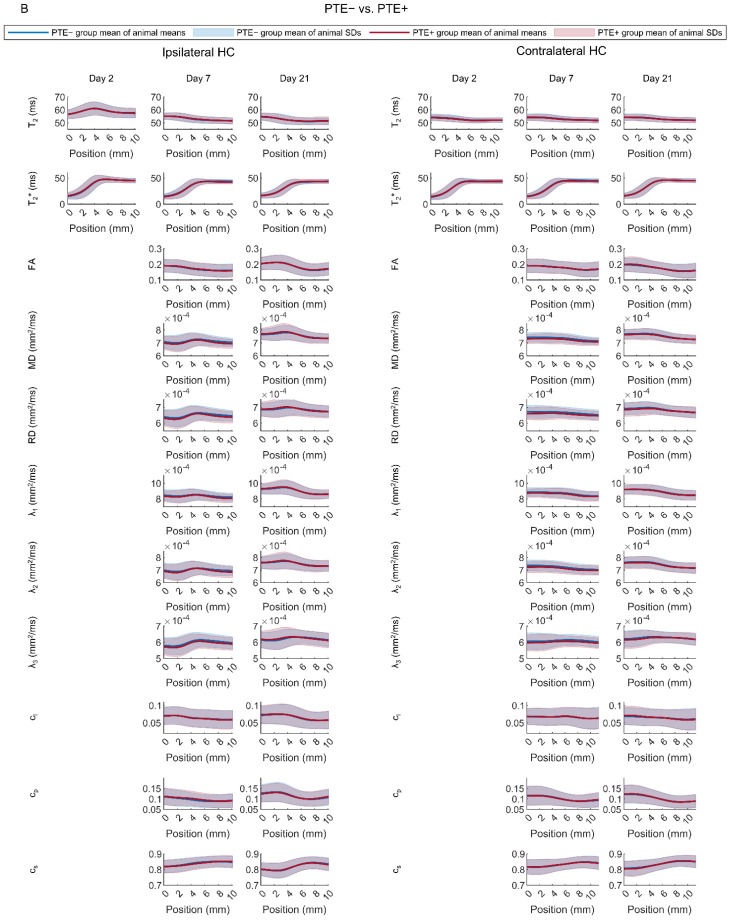

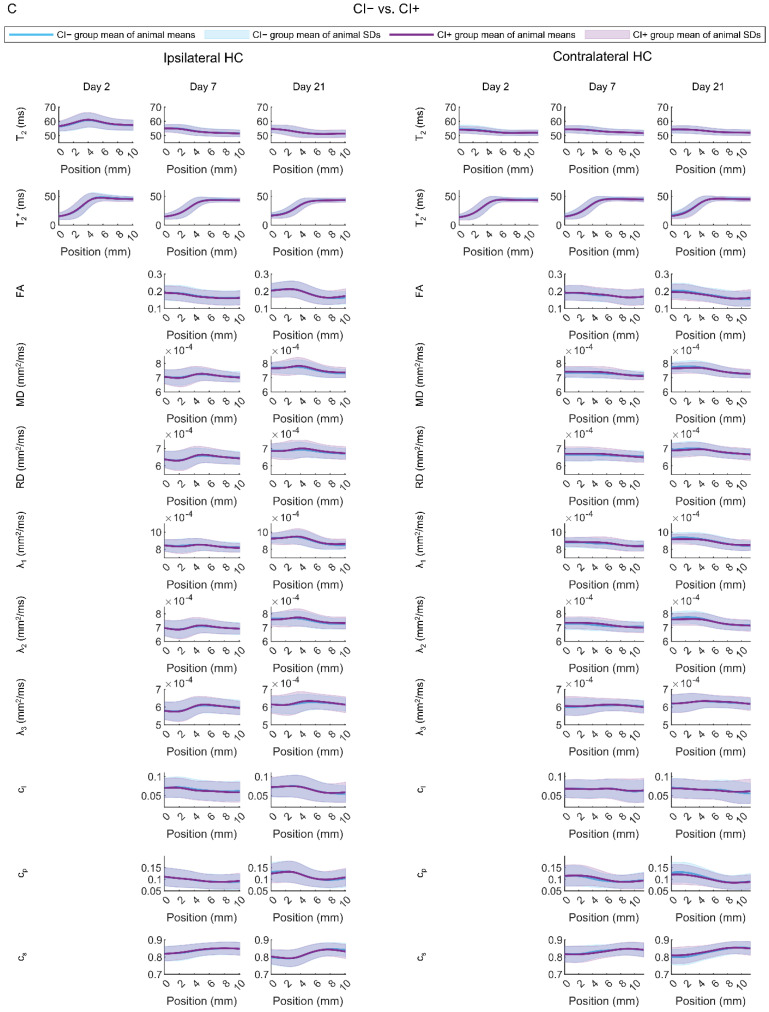
Hippocampal MRI parameter maps. (**A**) Comparison between rats with sham-operation or traumatic brain injury (TBI). (**B**) Comparison between injured rats without (TBI−) or with (TBI+) epilepsy. (**C**) Comparison between injured rats without (CI−) or with (CI+) cognitive impairment. The mean and standard deviation (SD) of each MRI parameter map were estimated at different positions along the septotemporal axis of the hippocampus for each animal (0 refers to the temporal pole). The plots show the group averages of the animal means (line) and SDs (shaded area). Abbreviations: FA, fractional anisotropy; MD, mean diffusivity; RD, radial diffusivity. Statistical significance: The Mann–Whitney U-test was used to test for differences between the groups in mean and SD of each parameter at each position along the hippocampal axis. A statistically significant difference after controlling for multiple comparisons is indicated with *.

First, we investigated the differences in the distributions of individual MRI parameters along the septotemporal axis of the ipsilateral and contralateral hippocampus between the sham and TBI groups (Figure 5A).

We detected group differences along the ipsilateral hippocampus on all analysis days (Mann–Whitney U test, false discovery rate-corrected q < 0.05). On D2, the sham and TBI groups differed in the mean T_2_ and T_2_*. On D7, they differed in the mean T_2_, T_2_*, FA, MD, RD, λ_1_, λ_2_, λ_3_, c_l_, c_p_, and c_s_. On D21, we found group differences in the mean T_2_, T_2_*, FA, RD, λ_2_, c_l_, c_p_, and c_s_. In addition, there were differences in the SD of T_2_ and T_2_* on D2, SD of T_2_, T_2_*, FA, MD, RD, c_l_, λ_1_, λ_2_, λ_3_, and c_s_ on D7, and SD of T_2_, T_2_*, FA, MD, RD, λ_1_, λ_2_, λ_3_, c_l_, c_p_, and c_s_ on D21 (Mann–Whitney U test, false discovery rate-corrected q < 0.05).

Furthermore, along the contralateral hippocampus, we detected group differences at all three time points (Mann–Whitney U test, false discovery rate-corrected q < 0.05). On D2, the groups differed in the mean T_2_ and T_2_*. On D7, there were group differences in the mean T_2_, T_2_*, FA, λ_1_, λ_2_, c_l_, and c_s_. On D21, a group difference was found in the mean T_2_*, FA, c_l_, and c_s_. There were also group differences in the SD of T_2_* on D2, SD of FA, λ_3_, c_l_, and c_s_ on D7, and SD of T_2_*, FA, c_l_, c_p_, and c_s_ on D21.

We computed the area under the receiver-operating characteristic curve (AUC) for each parameter at a given position. In the ipsilateral hippocampus, the mean of T_2_ on D2 at position 2 mm from the temporal end was able to distinguish between the sham and TBI animals with an AUC value of 1.0 (95% confidence interval 1.0–1.0). In the contralateral hippocampus, the SD of FA on D21 at position 11 mm from the temporal end (being close to the septal end) differentiated between the sham and TBI animals with an AUC value of 0.84 (95% confidence interval 0.74–0.91).

##### Rats with (TBI+) vs. Those without (TBI−) Post-Traumatic Epilepsy

No statistically significant differences were found in the distributions of any of the single MRI parameters between the TBI− and TBI+ groups (Figure 5B) (Mann–Whitney U test, false discovery rate-corrected q > 0.05). In the ipsilateral hippocampus, the highest AUC value for distinguishing between the TBI− and TBI+ animals was 0.70 (95% confidence interval 0.52–0.83) for mean T_2_* at the septal end on D7. In the contralateral hippocampus, the highest AUC value was 0.71 (95% confidence interval 0.52–0.85) for SD of λ_1_ at the temporal pole on D21.

##### Rats with (CI+) vs. Those without (CI−) Cognitive Impairment

No statistically significant differences were found in the distributions of any of the single MRI parameters between CI− and CI+ groups (Figure 5C) (Mann–Whitney U test, false discovery rate-corrected q > 0.05). In the ipsilateral hippocampus, the highest AUC value for distinguishing between the CI− and CI+ animals was 0.77 (95% confidence interval 0.61–0.88) for SD of λ_3_ at 9.6 mm from the temporal end on D21. In the contralateral hippocampus, the highest AUC value was 0.77 (95% confidence interval 0.58–0.89) for mean c_p_ at 5.4 mm from the temporal end on D7.

#### 3.2.2. Regularized Logistic Regression Analysis

**Figure 6:**
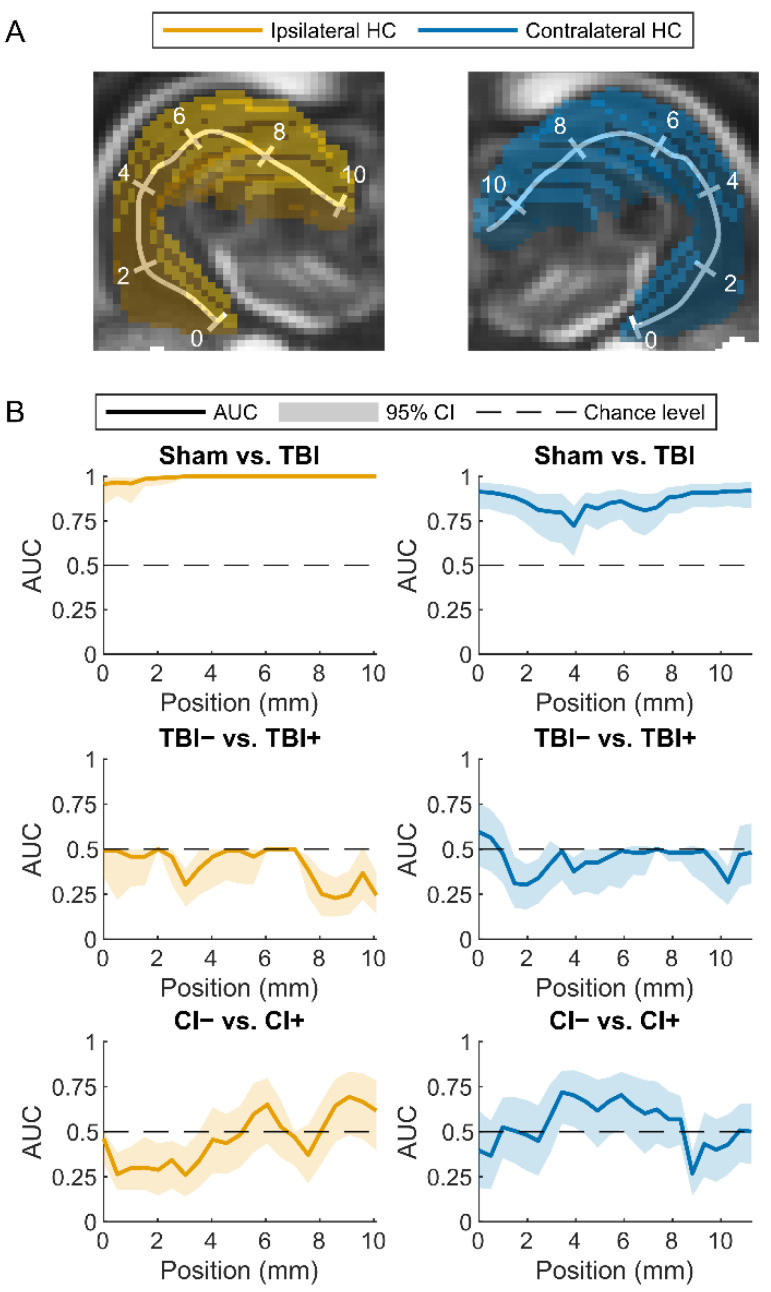
Classification performance of the elastic net-regularized logistic regression models. (**A**) Locations of analysis points along the ipsilateral (left, yellow) or contralateral (right, blue) hippocampus (HC). (**B**) Cross-validated area under the receiver operating characteristic curve (AUC) and its 95% confidence interval (95% CI) for different classification tasks: sham vs. TBI (traumatic brain injury), animals without (TBI−) or with (TBI+) epilepsy and rats without (CI−) or with (CI+) cognitive impairment. MRI parameters assessed in the ipsilateral hippocampus resulted in an almost perfect classification between the sham and TBI animals. The abnormalities in the temporal and septal ends of the contralateral hippocampus also resulted in an excellent separation of the sham from the TBI animals. Instead, ipsilateral or contralateral hippocampal abnormalities did not differentiate between the TBI− and TBI+ groups. Interestingly, the abnormalities in the contralateral hippocampus located at levels 3.4, 3.9, and 5.9 mm from the temporal end resulted in a lower bound of the confidence interval of AUC higher than 0.5 (AUC at 3.4 mm 0.72 with confidence interval 0.55–0.85, AUC at 3.9 mm 0.70 with confidence interval 0.53–0.84, AUC at 5.9 mm 0.70 with confidence interval 0.52–0.84), indicating that it may be possible to differentiate between animals without (CI−) and with (CI+) cognitive impairment.

As single hippocampal parameters performed poorly as biomarkers for post-traumatic epilepsy or cognitive impairment, we next assessed whether a combination of parameters would achieve a better performance. We fitted elastic net-based regularized logistic regression models to distinguish (a) TBI from sham animals, (b) TBI+ from TBI− animals, and (c) CI+ from CI− animals. Cross-validated AUC values computed from the fits at different positions along the septotemporal axis of the ipsilateral and contralateral hippocampus (Figure 6A) are shown in Figure 6B.

##### Sham vs. TBI

**Figure 7:**
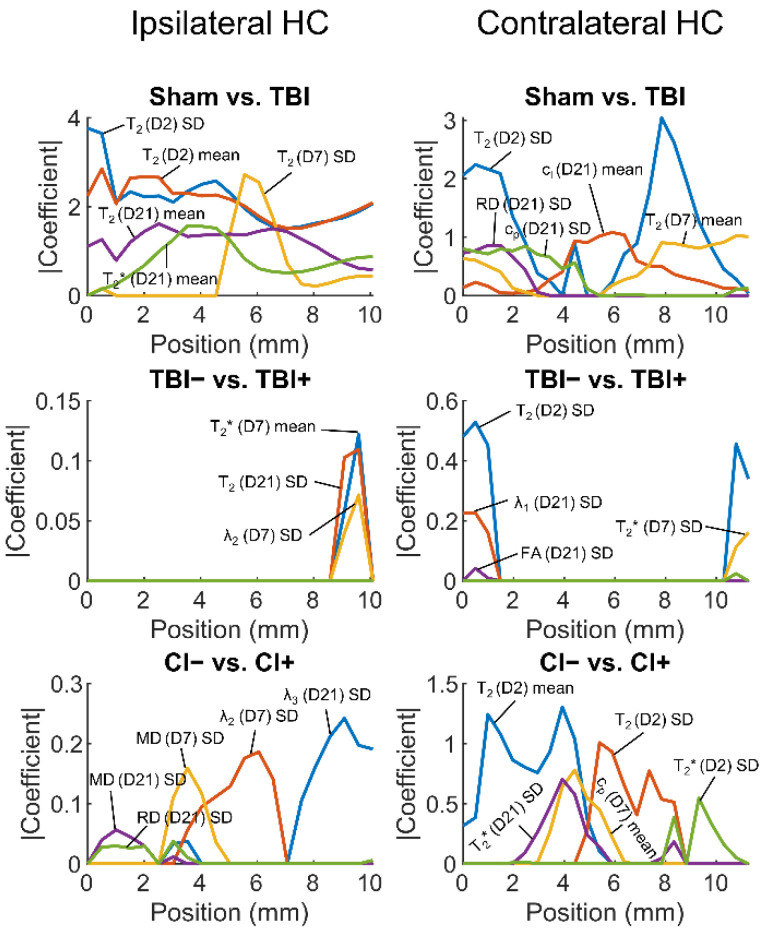
The most important predictors in logistic regression models. The predictors were normalized (mean 0, SD 1) before model fitting so that the absolute values of their estimated coefficients reflected their importance. Then, the estimated coefficients were averaged over the cross-validation folds. Five predictor variables with the highest absolute coefficients (*y*-axis) at any point along the hippocampus (*x*-axis) were selected. Abbreviations: CI−, rats without cognitive impairment; CI+, rats with cognitive impairment; D, day; FA, fractional anisotropy; HC, hippocampus; MD, mean diffusivity; RD, radial diffusivity; SD, standard deviation; TBI, traumatic brain injury; TBI−, rats without epilepsy; TBI+, rats with epilepsy.

A combination of parameters at positions more than 3 mm from the temporal end towards the septal end of the ipsilateral hippocampus differentiated TBI rats from sham-operated controls with an AUC value of 1.0 (95% confidence interval 1.0–1.0) (Figure 6 and Figure 7). The AUC value was high even at the temporal pole of the ipsilateral hippocampus (AUC 0.96, 95% confidence interval 0.85–0.99).

Surprisingly, the contralateral hippocampal parameters were able to differentiate between sham and TBI rats both at the temporal (AUC 0.91, 95% confidence interval 0.81–0.96) and septal ends (AUC 0.92, 95% confidence interval 0.82–0.97). Even the lowest AUC value at 3.9 mm from the temporal end of the contralateral hippocampus was moderate (AUC 0.72, 95% confidence interval 0.56–0.83).

##### Rats with (TBI+) vs. Those without (TBI−) Post-Traumatic Epilepsy

The hippocampal parameters did not differentiate TBI+ from TBI− rats at any location along the ipsilateral or contralateral hippocampus as the lower bound of the 95% confidence interval of the cross-validated AUC was smaller than 0.5. The highest AUC value (0.59, 95% confidence interval 0.40–0.75) was evident at the temporal end in the contralateral hippocampus.

##### Rats with (CI+) vs. Those without (CI−) Cognitive Impairment

In the ipsilateral hippocampus, CI+ rats could not be distinguished from CI− rats (lower bound of the 95% confidence interval of the cross-validated AUC smaller than 0.5). The highest AUC value (0.69, 95% confidence interval 0.48–0.84) was achieved at 9.1 mm from the temporal end.

In the contralateral hippocampus, however, the lower bound of the 95% confidence interval of the AUC was larger than 0.5 at 3.4, 3.9, and 5.9 mm from the temporal end of the hippocampus. This suggests that it may be possible to distinguish those rats with CI from those without any impairment. The highest AUC value (0.72, 95% confidence interval 0.55–0.84) was achieved at 3.4 mm from the temporal end.

#### 3.2.3. Volume of the Hippocampus

The distributions of hippocampal volumes in the different groups of animals are shown in Figure S5. A regularized logistic regression model that used the ipsilateral and the contralateral hippocampal volume at each time point as predictor variables differentiated TBI from sham animals (AUC 0.99, 95% confidence interval 0.96–1.0), but could not differentiate TBI+ from TBI− rats (AUC 0.59, 95% confidence interval 0.42–0.74), nor CI+ from CI− rats (AUC 0.56, 95% confidence interval 0.38–0.74).

#### 3.2.4. Summary of the Findings

**Figure 8:**
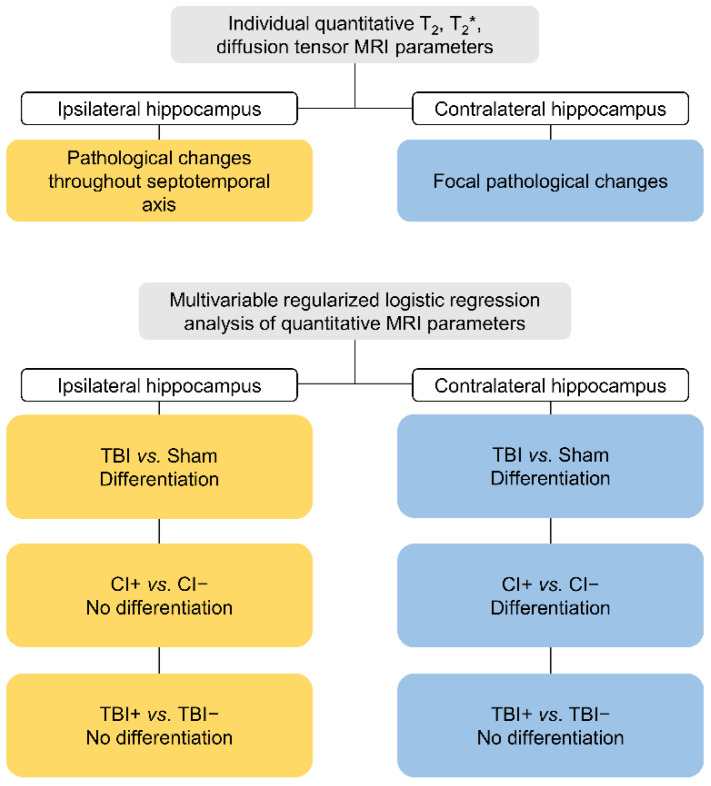
A summary of the main findings.

A diagram summarizing the main findings is presented in Figure 8.

## 4. Discussion

The hippocampus undergoes molecular and cellular pathologies over the weeks to months after a TBI in an experimental model as well as in humans suffering this kind of injury [34]. These abnormalities have been proposed to associate with the development of PTE and cognitive impairments [16,17]. The present study was designed to test a hypothesis that acute alterations in hippocampal MRI parameters can be used as prognostic biomarkers for epileptogenesis and the evolution of the cognitive decline.

### 4.1. Both Ipsilateral and Contralateral Hippocampal Abnormalities Differentiate TBI Animals from Sham-Operated Controls

The contribution of hippocampal pathology to epileptogenicity can differ along the septotemporal axis in experimental models and humans with epilepsy [18,35,36,37]. The septal (posterior hippocampus in primates) rather than the temporal (anterior) hippocampus appears to be more involved in the formation of spatial memory. Furthermore, epileptogenic injuries such as TBI can affect the hippocampus bilaterally [15,17]. Therefore, we quantified T_2_, T_2_*, and DTI MRI parameters along the septotemporal axis of both the ipsilateral and the contralateral hippocampus. At various times after the injury, we examined whether it would be possible to predict if an animal would develop post-injury epileptogenesis. Therefore, we determined MRI abnormalities at both acute and subacute post-TBI time points (D2, D7, D21 post-TBI). It is during this time frame that the initial hippocampal vasogenic edema evolves and then subsides, and several secondary injury mechanisms (e.g., apoptosis, axonal injury) become activated [34].

Interestingly, TBI rats exhibited pathological MRI features in both the ipsilateral and the contralateral hippocampus when compared to the sham-operated experimental controls. On D2 after TBI, we detected increased T_2_ values throughout the ipsilateral hippocampus. By D7, T_2_ had either normalized (temporal end) or decreased (septal end). By D21, T_2_ in the injured rats had decreased throughout the ipsilateral hippocampus except at its temporal end. This is indicative of the presence of vasogenic edema within the first days after TBI and its resolution thereafter. The decreased T_2_ values at D7 and D21 also suggest a reduced free water pool, possibly due to increased cellularity, or increased susceptibility differences due to iron accumulation.

We did not acquire diffusion data on D2 as we were worried about increased mortality at this early post-TBI time-point related to the lengthy anesthesia required in this imaging modality. On D7, mean diffusivity was increased throughout the septotemporal axis of the ipsilateral hippocampus, and returned to normal by D21. This could be a sign of the presence of cytotoxic edema still on D7 that had resolved by D21. The mean or variance of FA was decreased over distances of 1–5 mm from the septal end on D7; this is suggestive of interneuron loss [15,17]. On D21, the linear component of the diffusion tensor had decreased, and the planar component increased in the septal end of the ipsilateral hippocampus. This might reflect the emergence of differently oriented fibers, for example due to axonal sprouting, which shifted the mode of anisotropy from linear to planar in each imaging voxel [38].

An analysis of the contralateral hippocampus also demonstrated the presence of pathological features, which were, however, less pronounced than in the ipsilateral region. For example, on D7, we observed decreased T_2_ at the septal end. As speculated above for the ipsilateral hippocampus, this could be attributable to increased cellularity or the presence of iron or calcium accumulation. On D21, we observed decreased FA, a reduced linear diffusion component (c_l_), and increased spherical diffusion component (c_s_) at the septal end. These findings point to some degree of interneuron loss as observed in earlier studies [15,17]. The findings also suggest that—unlike in the ipsilateral hippocampus—there was no substantial axonal sprouting.

As revealed in the regularized logistic regression analysis, quantitative MRI was effective at distinguishing between sham and TBI rats, especially in the ipsilateral hippocampus. Interestingly, this technique also detected the presence of TBI-induced damage in the contralateral hippocampus; however, this was subtle, as indicated by the only slight differences in the individual MRI parameters between the sham and TBI rats. Importantly, in most cases, our multivariable regularized logistic regression analysis was able to distinguish between sham and TBI.

### 4.2. Contralateral Hippocampal Abnormalities Display a Moderate Performance in Differentiating Cognitively Impaired Animals from Those without a Cognitive Impairment

In the present animal cohort, 81% of the rats were cognitively impaired and 19% did not display any signs of cognitive impairment at 37 days after TBI as assessed using a cut-off point of 19.2 sec to find the hidden platform in the Morris water maze test. Interestingly, the multivariable regularized logistic regression MRI analysis along the septotemporal axis of the contralateral hippocampus distinguished between the cognitively impaired TBI rats and the animals with normal cognitive functioning. This finding agrees with previous studies that rats exhibited an impaired Morris water maze performance after a lateral FPI [14,15,39,40]. The impairment was already evident on D2 after the injury [14] and endured for up to one year [39]. This disturbed performance was related to both impaired learning as well as to memory retention [40]. In previous studies, impaired Morris water maze performance has been associated with hilar neuronal cell loss both in the ipsilateral and contralateral hippocampus [14,15].

Unexpectedly, MRI analysis of the contralateral but not the ipsilateral hippocampus differentiated between the TBI rats with and those without a cognitive impairment. However, the lower bounds of the 95% confidence interval of the ROC AUC values were close to being higher than 0.5 at the septal end of the ipsilateral hippocampus. The apparent failure of ipsilateral septal hippocampal damage to impact on the animals’ performance in the water maze could be related to the small sample size.

### 4.3. Hippocampal Abnormalities Do Not Differentiate Epileptic from Non-Epileptic Animals

The hippocampus has been claimed to be involved in epileptogenesis and ictogenesis in human patients and also in animal models of epilepsy [16,41]. We investigated whether our quantitative MRI analysis along the septotemporal axis of the hippocampus could identify the rats that would or would not develop post-traumatic epilepsy after a TBI. Although prior studies have associated pathological hippocampal features with post-traumatic epileptogenesis [18,41,42], neither individual MRI parameters nor regularized logistic regression models utilizing these parameters could identify the rats that would develop post-traumatic epileptogenesis.

### 4.4. Methodological Considerations

Progressively expanding abnormalities in internal structure, shape, and orientation of the hippocampus after lateral FPI complicate the image registration of the specific intrahippocampal subfields (dentate gyrus, CA3, CA2, CA1). This could have been further exacerbated by the low 0.5-mm rostral–caudal image resolution used. Therefore, we analyzed MRI measures in cross-sectional slices rather than in different hippocampal subfields along the septotemporal axis of the hippocampus. Consequently, based on the shape of the segmented hippocampi, we considered the gross hippocampus-to-hippocampus image registration to be sufficiently accurate.

An analysis of MRI hippocampal subfield abnormalities could help to detect associations between post-TBI abnormalities and epileptogenesis and/or cognitive impairment. However, this kind of approach would work best in those cases in which there was negligible hippocampal atrophy such as mild TBI. In the presence of substantial hippocampal atrophy, accurate segmentation or image registration of hippocampal subfields requires high image resolution and good contrast between the different subfields.

Our analysis included MRI data acquired between 2 and 21 days after TBI, i.e., the optimal time period for the initiation and progression of secondary pathologies [34]. However, as the exact time of post-traumatic epileptogenesis remains to be determined, an analysis of hippocampal MRI parameters at more chronic post-TBI time points could produce valuable information for predicting the emergence of epilepsy.

## 5. Conclusions

After an experimental TBI, quantitative multiparametric MRI of the hippocampus can be used to differentiate rats that will develop cognitive impairments from those that will remain cognitively normal. The prognostic biomarker parameter set could be used to enrich a study cohort for interventions aimed at restoring memory functions. However, the analysis could not differentiate between the rats that would develop post-traumatic epilepsy and those not showing evidence of late spontaneous seizures.

## Data Availability

The data presented in this study are available on request from the corresponding author.

## Supplementary Materials

The following supporting information can be downloaded at: https://www.mdpi.com/article/10.3390/biomedicines10112721/s1, Supplementary methods; Figure S1: Examples of exclusions due to poor hippocampal segmentation; Figure S2: Exclusions due to poor image registration results; Figure S3: Hippocampal outlining and artefact removal; Figure S4: Examples of image artefact removal in the hippocampus; Figure S5: Volume of the hippocampus in the different groups of animals.
