# Supplementary material for: Acute Hippocampal Damage as a Prognostic Biomarker for Cognitive Decline but Not for Epileptogenesis after Experimental Traumatic Brain Injury
Source: Biomedicines. 2022 Oct 27;10(11):2721. doi: 10.3390/biomedicines10112721 (PMC9687561; doi:10.3390/biomedicines10112721)
Supplement: Supplementary file 1 [file biomedicines-10-02721-s001.zip › biomedicines-1968274-supplementary.pdf]

Supplementary methods

The MRI analysis was conducted in the template space, to which all the animals had been transformed using image registration. The hippocampal borders could be affected by a partial volume effect as well as image registration inaccuracies, introducing signals not originating from the hippocampus. Thus, we applied an image erosion operation (MATLAB function “imerode”, using a disk element with a two-pixel radius) at each hippocampal slice to remove the border region (Figure S3A).

Some of the diffusion images contained image artefacts within the hippocampus. We devised a method to remove the image voxels corrupted by these artefacts. First, we computed the mean fit error (mean residual) for each voxel. Then, we computed the fit error mean and SD over all voxels among the animals that showed no visible image artefacts. We defined any imaging voxel with a fit error higher than (mean + 2*SD) as a voxel contaminated by image artefacts and removed it (Figure S3B). Finally, we visually inspected the success of our image artefact removal procedure and excluded animals in which it was deemed unsuccessful. Examples of unsuccessful artefact removal are shown in Figure S4.

Four of the most caudal hippocampal slices exhibited a low mean signal-to-noise ratio, resulting in a high mean proportion of voxels being removed per animal (Figure S3C). To increase the accuracy of the analysis, we removed slices with >20% mean proportion of removed voxels. This meant that slices starting at the rostrocaudal level -5.5 mm from bregma were removed from the analysis (Figure S3D).

Supplementary Figures

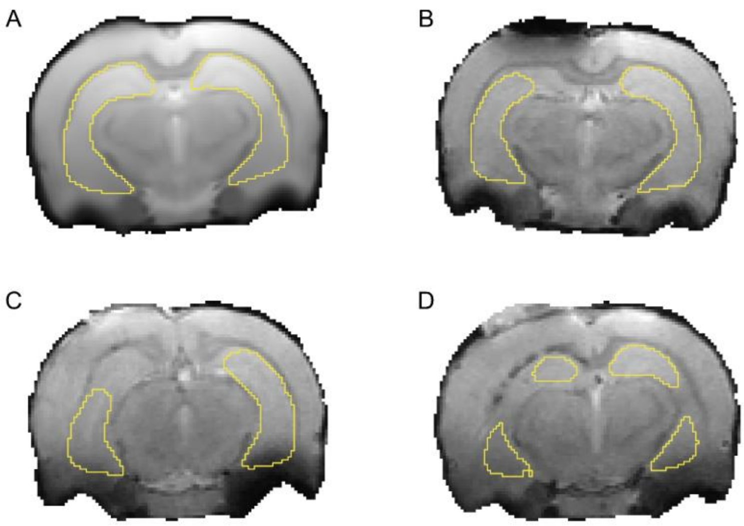

Figure S1. Examples of exclusions due to poor hippocampal segmentation. **(A)** Accurate hippocampal segmentation in the template brain. **(B)** A poor segmentation result in the ipsilateral hippocampus, likely due to variations in the image intensity resulting from a magnetic field inhomogeneity caused by the craniotomy. Poor hippocampal segmentation was most common in animals with variations in the visible image intensity near the craniotomy. **(C)** A poor segmentation result in the ipsilateral hippocampus due to some unknown reason. **(D)** A poor segmentation result in the ipsilateral and contralateral hippocampus.

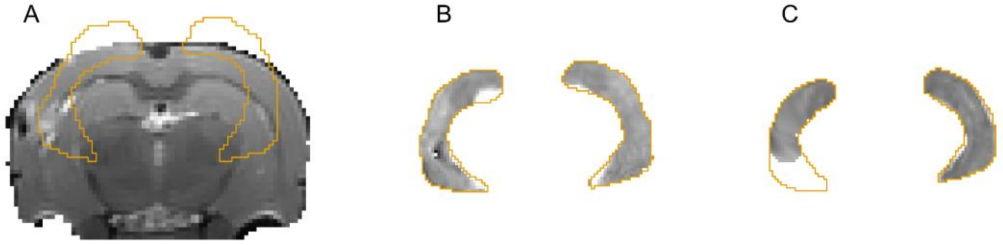

Figure S2. Exclusions due to poor image registration results. **(A)** In one case, a poor image registration from gradient echo images to spin echo images caused a severe misalignment of the hippocampal segmentation in the spin echo images. **(B)** In one case, a poor image registration from gradient echo images to diffusion-weighted images caused the hippocampal region of interest to contain a considerable amount of cerebrospinal fluid. **(C)** In one case, the presence of considerable hippocampal atrophy at 21 days after the injury resulted in a poor image registration.

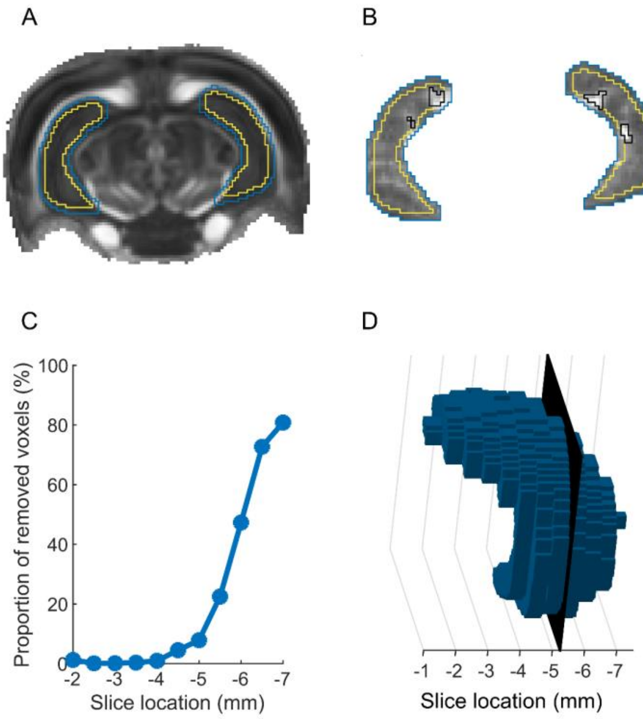

Figure S3. Hippocampal outlining and artefact removal. **(A)** To reduce the influence of image registration inaccuracy and remove the partial volume effect in the hippocampal border areas, a 2-voxel-wide layer (see Methods) was peeled-off from the hippocampal surface (from blue to yellow outline) in each slice. **(B)** Within the hippocampus, the mean fit error was computed for each voxel to detect and remove those voxels which were contaminated by image artefacts (black outline). **(C)** The proportion of removed voxels for each slice location averaged over animals. The most caudal slices in the diffusion imaging sequence had low signal-to-noise ratios, resulting in a high mean proportion of removed voxels. Therefore, slices starting at the rostrocaudal level -5.5 mm from bregma (mean proportion of removed voxels >20%) were removed from the analysis. **(D)** Sagittal view of the hippocampal surface. The slices behind the black plane were removed from the analysis.

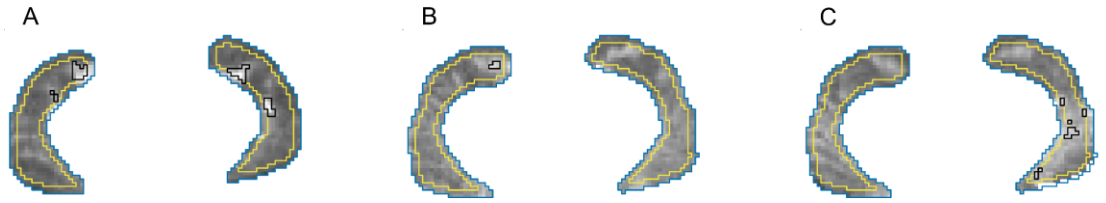

Figure S4. Examples of image artefact removal in the hippocampus. Grayscale intensity describes the mean fit error for each imaging voxel. Image voxels with high mean fit error (black outline) within the analyzable hippocampal region (yellow outline) were excluded. The success of the removal was visually assessed in each animal. **(A)** An example of a successful artefact removal. **(B, C)** Examples of unsuccessful artefact removals.

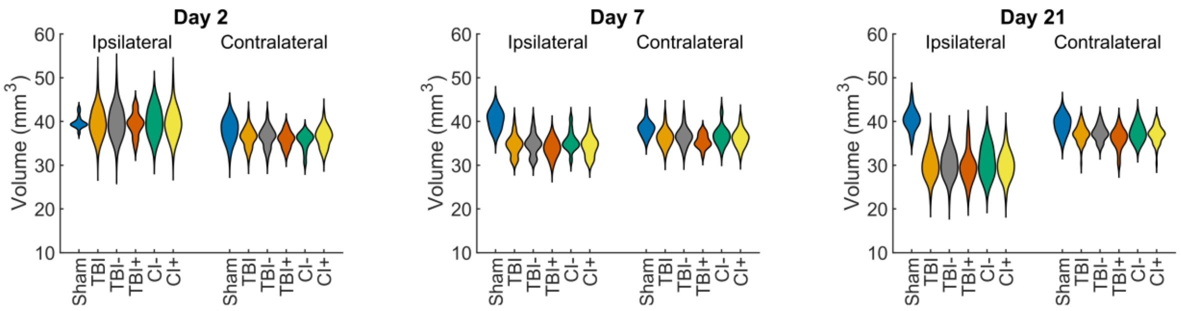

Figure S5. Volume of the hippocampus in the different groups of animals. The ipsilateral hippocampus underwent progressive atrophy in the rats with traumatic brain injury (TBI) as compared to those rats that underwent a sham operation. No differences were found between the TBI rats that developed epilepsy (TBI+) and those that did not develop epilepsy (TBI-), nor between the TBI rats that had a cognitive impairment (CI+) and those that did not have a cognitive impairment (CI-).
